# Evaluation of Yeast Fermented Poultry By-Product Meal in Nile Tilapia (*Oreochromis niloticus*) Feed: Effects on Growth Performance, Digestive Enzymes Activity, Innate Immunity, and Antioxidant Capacity

**DOI:** 10.3389/fvets.2019.00516

**Published:** 2020-01-24

**Authors:** Mahmoud A. O. Dawood, Fawzy I. Magouz, Mohamed Mansour, Ahmed A. Saleh, Amel M. El Asely, Sabreen E. Fadl, Hamada A. Ahmed, Khalid A. Al-Ghanim, Shahid Mahboob, Fahad Al-Misned

**Affiliations:** ^1^Department of Animal Production, Faculty of Agriculture, Kafrelsheikh University, Kafr El Sheikh, Egypt; ^2^Department of Poultry Production, Faculty of Agriculture, Kafrelsheikh University, Kafr El Sheikh, Egypt; ^3^Department of Aquatic Animals Diseases and Management, Faculty of Veterinary Medicine, Benha University, Benha, Egypt; ^4^Biochemistry Department, Faculty of Veterinary Medicine, Matrouh University, Matrouh, Egypt; ^5^Department of Nutrition and Veterinary Clinical Nutrition, Faculty of Veterinary Medicine, Damanhour University, Damanhour, Egypt; ^6^Department of Zoology, College of Science, King Saud University, Riyadh, Saudi Arabia

**Keywords:** digestive enzymes, immunity, Nile tilapia, oxidative status, poultry by product meal, yeast fermentation

## Abstract

The aim of the present study was to examine the effects of dietary inclusion of fermented poultry by-product meal (FPBM) on growth performance, digestive enzymes activity, innate immunity, and antioxidant capacity in Nile tilapia (*Oreochromis niloticus*). A basal diet containing fish meal and soybean meal was considered as a control (Con), and four other diets were produced by inclusion of 10, 20, 30, or 40% FPBM (FPBM10, FPBM20, FPBM30, and FPBM40 diets). The experiment was done in triplicates (20 fish per replicate) and the fish were fed the test diets to visual satiety twice daily for 8 weeks. The groups of fish fed the FPBM10 and FPBM20 diets showed significantly (*P* < 0.05) higher weight gain and specific growth rate, and lower feed conversion ratio than those fed the Con and FPBM40 diets. Moreover, inclusion of 40% FPBM led to significant reduction of feed intake compared to the other treatments. FPBM at all the tested levels improved intestinal protease activity and lipase activity was enhanced at 10–30% inclusion levels. Furthermore, the FPBM10 and FPBM20 groups revealed significantly higher amylase activity than the other treatments. The FPBM10 group exhibited significantly higher phagocytic activity than the control group and phagocytic index was enhanced by dietary inclusion of 10–30% FPBM. However, inclusion of over 30% FPBM led to significant reduction of lysozyme, phagocytic, and bactericidal activities compared to the control group. Further, FPBM10 and FPBM20 diets increased the serum IgM levels, while NBT was significantly increased by feeding FPBM10 diet compared with FPBM30 and FPBM40 groups (*P* < 0.05). The group fed the FPBM30 diet showed significantly higher glutathione peroxidase activity than the control group. According to the analysis of the data by the polynomial regression, the inclusion of FPBM at 11.17–25.14% can be applied effectively in the diets of tilapia for better growth performance and health condition.

## Introduction

The global supply of fish for human consumption has outpaced population growth in the past five decades (1). This has raised attention to increasing the development of the aquaculture industry leading to advanced production technologies and culture systems worldwide (2). Therefore, the industry would soon run out of sufficient quantities of fish oil and fishmeal (FM) (3, 4). As a result of increasing demand, limited supply, and a dramatic increase in FM price, suitable alternative protein sources for fish feed have recently been intensively studied (5). Any reduction in feed costs with a preserved health status of fish is bound to have a direct positive effect on the profitability of aquaculture.

The use of protein sources of animal origin as FM replacement in fish feed is a growing trend in the aquaculture industry (6). In this context, among commercially available animal protein alternatives, poultry by product meal (PBM) has been one of the best nutritional value and amino acid balances (lipids, 12–15% and proteins, 58–65%), except for the low level of lysine and methionine (7, 8). It is widely available at competitive prices and is therefore one of the main dietary protein alternatives to FM in the feed of cultured species (9–11). Potential problems in PBM feeding exist due to the existence of fibers and high levels of lipids, which can cause high oxidation, malnutrition, and lower palatability (12, 13). To reduce the costs of the feed and environmental pollution, PBM can be treated with suitable microorganisms with beneficial effects (14, 15). Recently, the fermentation strategy has been applied to balance the nutritional value of animal protein sources (16). The microorganisms can be active under anaerobic conditions in order to dissimilate the ingredients organic contents (5). By this way the long chain amino acids and fatty acids can be shortened to be more available for absorption in the gastrointestinal tract of fish (17–19). It has been reported that the animal proteins (e.g., poultry feather meal, fish meal, and animal protein blend) fermented with various microorganisms such as fungus (*Aspergillus* sp.), bacteria (*Lactobacillus* sp.), and yeast (*Saccharomyces cerevisiae*) shown to have an enhanced nutritional status in terms of higher protein and lower fiber fractions in comparison to the respective untreated ones (5, 16, 20).

Though fermented animal proteins showed better performance than the respective untreated ones, still their inclusion level by substituting FM is not yet at the satisfied level. Samaddar et al. (16) concluded that there was no negative effect on growth of *Labeo rohita* when dietary FM was substituted up to 75% using a fermented animal protein blend. Mondal (20) was also able to substitute 75% FM with fermented PBM (FPBM) in the diet of Indian major carp (*Catla catla*). To date, no data available about using FPBM on the growth performance and health condition of Nile tilapia (*Oreochromis niloticus*).

Based on records of fish growth, palatability, digestive enzymes activity, apparent digestibility coefficients of nutrients, nutrient retention, blood biochemistry, and histopathologic aspects (21–23), FPBM appears to attribute benefits on fish quality and nutrients retention efficiency without indications of negative physiological impacts for the fish in short term studies. As a principal species, Nile tilapia is cultured widely as the second major candidate for aquaculture (24). Though the productivity of tilapia is rapidly increasing, profitability of the tilapia production is being decreased due to the progressive increase in feed cost (25, 26). Thus, the present study was conducted to evaluate the effects of dietary inclusion levels of FPBM on growth performance, digestive enzymes activity, and immunity of Nile tilapia.

## Materials and Methods

The experimental procedure was approved by the Institutional Animal Care and Use Committee in Kafrelsheikh University (Kafrelsheikh, Egypt).

### Fermentation and Diet Preparation

Fine powder of PBM was kindly provided by Elsodor company (Al-Sadat city, Egypt). PBM was adjusted to a moisture content of 60–65% using de-ionized water in a 1 L glass container with six sets of replications for each. They were autoclaved at 121°C (105 kPa) (20 min) then cooled to room temperature. Cane molasses was collected from the local market and used as a source of fermentable carbohydrates for *S. cerevisiae* activation. To the sterilized ingredients, actively growing culture (2.27 × 10^10^ CFU per g) of *S. cerevisiae* (Shanghai Gosun Biotechnologies CO. LTD, China) was inoculated and thoroughly mixed using a sterile glass rod manually. All the inoculated samples were incubated at 30 ± 2°C for 4 days to carry out the fermentation process. Prior to this, the fermentation conditions were optimized for moisture (50, 55, 60, 65, and 70%), temperature (28, 30, 32, and 36°C), inoculums rate (1, 2.5, 5, 7.5, and 10%), pH (5.5, 6, 6.5, 7, 7.5), and incubation period (1, 2, 3, 4, and 5 days). The optimized conditions of 60–65% moisture, 28–30°C temperature, 5% inoculum rate, 6.5–7 pH, and 4-day incubation period were applied in the present investigation. All the fermentation samples were mixed with a sterile glass rod two times every day (7.00 a.m. and 6.30 p.m.) for proper homogenization. After the fourth day, all the materials were transferred to a little higher temperature of 35°C to stop the fermentation process and allow to dry at the same temperature until the moisture content of fermented samples become <10%. The proximate and essential amino acid composition of FPBM is given in **Table 2**.

Five isonitrogenous (30.8% crude protein) and isolipidic (6.5% crude lipid) diets were formulated as shown in Table 1. The experimental diets were formulated to contain 0, 10, 20, 30, and 40% FPBM (Con, FPBM10, FPBM20, FPBM30, and FPBM40 diets) and were further balanced for crude protein, using FM, soybean meal, and other plant protein mixes (Table 1). Methionine, lysine, threonine, and tryptophan were added to obtain an equivalent indispensable amino acid profile. All ingredients were completely mixed, then added to produce a stiff dough and pelleted in a tabletop pelletizer with a 1–2 mm die, then the pellets were air dried. The diets obtained were stored at −20°C until they were used.

**Table 1:** Formulation of the experimental diets (%) used to fed Nile tilapia with varied levels of FPBM for 60 days.

**Ingredient**	**FPBM (%)**				
	**0**	**10**	**20**	**30**	**40**
Fish meal^a^	18	11	8	5	2
Soybean meal^a^	32	30	20	10	0
Fermented poultry by-product^b^	0	10	20	30	40
Corn gluten^a^	4	2	2	2	2
Wheat bran^a^	11	13	17	21	24
Rice bran^a^	12	12	12	12	12
Yellow corn^a^	15	15	15	15	15
Fish oil^a^	2	2	2	1	1
Sunflower oil^a^	3	2	1	1	1
Vitamin mixture^c^	1	1	1	1	1
Vitamin mixture^d^	1	1	1	1	1
Di-calcium phosphate^e^	1	1	1	1	1
Threonine^e^	0	0.04	0.12	0.16	0.22
Lysine^e^	0	0.15	0.36	0.5	0.68
Methionine^e^	0	0.05	0.11	0.16	0.21
Tryptophan^e^	0	0.06	0.16	0.23	0.31

a *Supplied by Feed Control Co., Ltd. (Damro, Sidi Salem, Kafrelsheikh, Egypt); fish meal, 66% crude protein, 7.9% crude lipid; soybean meal, 45.6% crude protein, 0.7% crude lipid; corn gluten, 60% crude protein, 2.1% crude lipid; wheat bran, 12.8% crude protein, 3.6% crude lipid; rice bran, 12.5% crude protein, 5.2% crude lipid; yellow corn, 7.5% crude protein, 3.5% crude lipid; Fish oil and sunflower oil (98–99% crude lipid)*.

b *Poultry by-product meal was kindly provided by Elsodor company (Al-Sadat city, Egypt); fish meal, 60.2% crude protein, 7.9% crude lipid*.

c *Vitamin mixture (mg/kg premix): vitamin A (3,300 IU), vitamin D_3_ (410 IU), vitamin E (2,660 mg), vitamin B_1_ (133 mg), vitamin B_2_ (580 mg), vitamin B_6_ (410 mg), vitamin B_12_ (50 mg), biotin (9,330 mg), colin chloride (4,000 mg), vitamin C (2,660 mg), inositol (330 mg), para-amino benzoic acid (9,330 mg), niacin (26.60 mg), pantothenic acid (2,000 mg)*.

d *Mineral mixture (mg/kg premix): manganese (325 mg), iron (200 mg), copper (25 mg), iodine, cobalt (5 mg)*.

e *Dicalcium Phosphate, methionine, L-lysine, threonine, tryptophan (DSM in Animal Nutrition and Health, Heerlen, the Netherlands)*.

### Fish and Experimental Protocol

Fingerlings of Nile tilapia were obtained from a local farm (Kafrelsheikh, Egypt), and transported to Animal Production Department, Faculty of Agriculture, Kafrelsheikh University (Kafrelsheikh, Egypt). Fish was acclimatized for 2 weeks then distributed (10.6 ± 0.3 g) into 15 glass aquaria (60 L) at stocking density of 20 fish per aquarium. All the experimental aquaria were fixed with continuous aeration through single air-stone. The feeding rate was 3% of fish weight and the respective test diets were provided twice daily (8 and 15:30) for 60 days (28). After feeding time, the uneaten feed, if any were removed and 50% of water was replaced with freshly dechlorinated water. The water quality parameters including temperature, dissolved oxygen, pH, and total ammonia were carefully monitored and maintained at 24.2 ± 1.6°C, 66.32 ± 0.3 mg/L, 7.05 ± 0.6, and 0.32 ± 0.01 mg/L, respectively.

### Sample Collection

All fish were fasted 24 h before the sampling. Before sampling, all fish were anesthetized with tricaine methane sulphonate (MS-222) at 25 mg/L, after which they were counted and weighed for growth and biometric indexes. Fish were measured individually for the final body weight and length. Blood samples were collected from the caudal vein of Nile tilapia (3 fish/aquarium) and pooled together. A portion of the blood was put into EDTA coated vials for whole blood and another part in non-coated vials for serum collection. Serum samples were separated by centrifugation of the coagulated blood at 3,000 rpm for 15 min and stored at −20°C for further analysis.

The whole intestine was then sampled from nine fish/group in an ice bath for the analysis of digestive enzymes. The collected intestine samples were pooled and immediately homogenized with cold PBS (pH 7.5; 1 g per 10 mL) and centrifuged at 8,000 ppm for 5 min at 4°C and the supernatants were kept at −80°C until assayed.

### Chemical Analysis

AOAC (29) standard method was used to confirm the nutritional profile of each diet, PBM, and FPBM (Table 2). Amino Acid Analyzer (Biochrom 30) used for identifying the amino acid profile (EAA) by following the protocol of the manufacturer.

**Table 2:** Proximate composition (%, dry matter basis) of PBM, FPBM, and the experimental diets (%) used to fed Nile tilapia with varied levels of FPBM for 60 days.

**Item**	**PBM**	**FPBM**	**FPBM (%)**					**Requirements^b^**
			**0**	**10**	**20**	**30**	**40**	
Crude protein	60.2	65	31.38	31.32	31.54	31.76	31.88	
Ether extract	7.9	8.1	6.04	6.3	6.46	6.55	6.54	
Ash	10.6	4.6	7.23	7.45	7.65	7.16	7.21	
Gross energy (kcal/g)^a^	470.68	508.73	445	451	449	448	446	
**Essential amino acid**								
Arginine	6.6	6.9	2.72	2.63	2.67	2.65	2.63	1.18
Histidine	1.8	2.1	1.12	0.96	0.95	0.89	0.88	0.48
Isoleucine	3.9	4.2	1.32	1.32	1.21	1.18	1.13	0.78
Leucine	7	7.3	2.04	2.02	2.14	2.31	2.43	0.95
Lysine	4.2	4.7	2.48	2.33	2.26	2.25	2.31	1.43
Methionine	1.22	1.7	1.41	1.25	1.18	1.21	1.23	0.75
Phenylalanine	3.7	4.2	0.95	0.93	0.89	0.91	0.89	1.05
Threonine	3.65	4.2	2.28	2.11	2.08	2.21	2.12	1.05
Tryptophan	0.7	1	1.01	0.95	0.93	0.94	0.92	0.28
Valine	5.22	5.7	0.96	0.92	0.89	0.82	0.85	0.78

a *Gross energy was calculated as 5.65, 9.45, and 4.11 kcal per g for protein, lipid, and carbohydrates, respectively*.

b *Nile tilapia requirements based on the recommendations of National Research Council (27)*.

### Growth Performance Calculations

All fish per tank were weighed and counted separately during the final sampling.

### Digestive Enzymes Activity

The total protein content was measured by Lowry et al. (30) method, in which BSA was used as a standard. According to Anson (31), Folin and Ciocalteus Phenol Reagent was used for measuring protease activity and iodine solution for measuring amylase activity to detect non-hydrolyzed starch according to Jiang (32) and Worthington (33). The protease and amylase activities were expressed as “U per mg of protein.” On the basis of the protocol described by Borlongan (34) and Jin (35) with olive oil as substrate, the specific activity of lipase was assessed. The activity of lipase was expressed as intestinal content “U per g intestine content.”

### Immunological Assays

The RA-50 chemistry analyzer (Bayer) used the total serum protein using ready-made kits of Spinreact Company Spain. ELISA kit of Cusabio; Wuhan, Hubei, China was used for immunoglobulin M (IgM) determination. The IgM result was expressed in mg per dl. According to Secombes (36), the nitro-blue-tetrazolium (NBT) was used for the detection of the respiratory burst activity of the blood using a micro-plate reader (Optica, Mikura Ltd., UK) at 630 nm.

The activity of lysozyme was measured by following Parry et al. (37). The result was expressed as “a 0.001/min reduction in absorption.” Following Rainger and Rowley (38), serum bactericidal activity against *Aeromonas hydrophila* was detected. As a survival index (SI), the results were recorded by this equation.

SI = CFU at the end/CFU at the beginning/100.

By following Kawahara et al. (39), the phagocytic activity and phagocytic index were determined by these equations:

Phagocytic activity = macrophages containing yeast/macrophages total number × 100.

Phagocytic index = phagocytized cells number/phagocytic cells number.

### Oxidative Status

Serum activities of antioxidant enzymes [superoxide dismutase (SOD), catalase (CAT), and glutathione peroxidase (GPx)] and concentration of lipid peroxidation [malonaldehyde (MDA)] were determined by using kits of Cusabio Biotech Co., Ltd; China.

### Statistical Analysis

All data obtained were analyzed by using one-way ANOVA SPSS version 22 except quadratic effects of various dietary FPBM levels on the observed response variables by polynomial contrasts and the optimum FPBM level by polynomial regression analysis (40).

## Results

### Nutrient Composition of PBM, FPBM, and Formulated Diets

Data of PBM, FPBM, and formulated diets analysis (Table 2) revealed that crude protein and ether extract contents are higher in FPBM (65 and 8.1%) compared to non-fermented PBM (60.2 and 7.9%). Similar trend was observed for gross energy between FPBM and non-fermented PBM and was in the range of 470.68–508.73 kcal/g. Though experimental diets were formulated to be balanced in protein (31.32–31.88%) and lipid (6.04–6.55%), the analyzed essential amino acids showed relatively higher values in FPBM compared to non-fermented PBM. The dietary change had a slight deviation in essential amino acids quantity, the nutritional requirement of Nile tilapia in essential amino acids was fulfilled even at higher substitution of FM in our study.

### Tilapia Growth Performance and Biometrics

All growth performance parameters demonstrated in Table 3 where rates of the survival were 93.3 and 98.6% without significant (*P* > 0.05) alterations. The condition factor also was not significantly affected by the inclusion of FPBM (*P* > 0.05).

**Table 3:** Growth performance and nutrient utilization of Nile tilapia fed FPBM diets for 60 days^*^.

**Item**	**FPBM (%)**					***P* value**
	**0**	**10**	**20**	**30**	**40**	
IBW (g)	10.6 ± 0.00	10.6 ± 0.02	10.6 ± 0.02	10.5 ± 0.01	10.6 ± 0.00	0.17
FBW (g)	40.9 ± 0.39^a^	44.4 ± 0.88^b^	45.2 ± 0.95^b^	43.1 ± 0.58^ab^	40.5 ± 0.52^a^	0.003
WG (%)	286 ± 3.70^a^	318 ± 8.60^b^	327 ± 9.77^b^	308 ± 5.20^ab^	281 ± 4.93^a^	0.003
SGR (% IBW/day)	2.25 ± 0.02^a^	2.39 ± 0.03^b^	2.42 ± 0.04^b^	2.34 ± 0.02^ab^	2.23 ± 0.02^a^	0.002
FI (g/fish/60 day)	41.4 ± 0.66^b^	41.9 ± 0.42^b^	42.1 ± 0.08^b^	41.5 ± 0.32^b^	39.4 ± 0.15^a^	0.005
FCR (g FI/g WG)	1.37 ± 0.04^c^	1.24 ± 0.02^ab^	1.22 ± 0.04^a^	1.28 ± 0.03^abc^	1.32 ± 0.02^c^	0.04
Survival (%)	94.6 ± 3.53	93.3 ± 3.53	98.6 ± 1.33	98.6 ± 1.33	93.3 ± 4.81	0.6
CF (%)	2.12 ± 0.21	2.27 ± 0.12	2.14 ± 0.15	2.18 ± 0.12	2.23 ± 0.22	0.26

* *Values expressed as means ± SE (n = 3). Different superscript letters indicate significant differences for each pairwise comparison between treatments. Weight gain (WG) = (FBW–IBW) × 100/IBW, specific growth rate (SGR) (BW/day) = 100 ((LnFBW–LnIBW)/T), feed efficiency ratio (FER) = WG/FI, feed conversion ratio (FCR) = FI/WG, survival = (final number of fish/initial number) × 100, condition factor (CF) = weigh of fish (g)/(length of fish)^3^ (cm)^3^ × 100, FBW, final body weight; IBW, initial body weight; T, trial duration in days; WG, weight gain; and FI, feed intake*.

It was observed that the inclusion of FPBM has a significant influence on fish performance parameters including FBW, WG, SGR, FI, and FCR where *P-*value was 0.003, 0.003, 0.002, 0.005, and 0.04, respectively (Table 3). The groups of fish fed the FPBM10 and FPBM20 diets showed significantly (*P* < 0.05) higher weight gain and specific growth rate, and lower feed conversion ratio than those fed the Con and FPBM40 diets. Moreover, inclusion of 40% FPBM led to significant reduction of feed intake compared to the other treatments. The growth performance and dietary FPBM levels were expressed by polynomial regression equations where, FBW (y = −0.0108x^2^ + 0.4121x + 41.113, *R*^2^ = 0.9699, optimal dose = 19.08%), WG (y = −0.1047x^2^ + 3.9733x + 287.63, *R*^2^ = 0.9795, optimal dose = 18.97%), SGR (y = −0.0004x^2^ + 0.0164x + 2.2576, *R*^2^ = 0.9829, optimal dose = 20.5%), and FCR (y = 0.0003x^2^ – 0.0127x + 1.3555, *R*^2^ = 0.9049, optimal dose = 21.17%) (**Table 7**).

### Digestive Enzymes Activity

FPBM at all the tested levels improved intestinal protease activity and lipase activity was enhanced at 10–30% inclusion levels (*P* < 0.05) (Table 4). Furthermore, the FPBM10 and FPBM20 groups revealed significantly higher amylase activity than the other treatments (*P* < 0.05). The lipase, amylase, and protease and dietary FPBM levels were expressed by regression equations (quadratic) where, amylase (y = −0.0108x^2^ + 0.377x + 31.913, *R*^2^ = 0.8208, optimal dose = 17.45%), lipase (y = −0.0318x^2^ + 1.1851x + 23.374, *R*^2^ = 0.8936, optimal dose = 18.63%), and protease (y = −0.0069x^2^ + 0.3469x + 29.239; *R*^2^ = 0.9711, optimal dose = 25.14%) (**Table 7**).

**Table 4:** Intestinal digestive enzymes activities of Nile tilapia fed FPBM diets for 60 days^*^.

**Item**	**FPBM (%)**					***P* value**
	**0**	**10**	**20**	**30**	**40**	
Lipase activity (U/g intestine)	22.1 ± 0.12^a^	34.5 ± 0.87^c^	34.6 ± 1.17^c^	27.5 ± 1.44^b^	21.3 ± 0.88^a^	0.04
Amylase activity (U/mg protein)	31.5 ± 0.47^a^	35.0 ± 0.97^b^	36.0 ± 1.15^b^	31.8 ± 0.20^a^	30.3 ± 0.47^a^	0.001
Protease activity (U/mg protein)	29.1 ± 0.48^a^	32.3 ± 0.86^b^	33.0 ± 0.78^b^	33.6 ± 0.30^b^	32.0 ± 0.68^b^	0.001

* *Values expressed as means ± SE (n = 3). Different superscript letters indicate significant differences for each pairwise comparison between treatments*.

### Immune Responses

The FPBM10 group exhibited significantly (*P* < 0.05) higher phagocytic activity than the control group and phagocytic index was enhanced by dietary inclusion of 10–30% FPBM (Table 5). However, inclusion of over 30% FPBM led to significant reduction of lysozyme, phagocytic, and bactericidal activities compared to the control group (*P* < 0.05). Further, FPBM10 and FPBM20 diets increased the serum IgM levels, while NBT was significantly increased by feeding FPBM10 diet compared with FPBM30 and FPBM40 groups (*P* < 0.05) (Table 5). However, the blood total protein was non-significantly affected by FPBM inclusion in tilapia diet (*P* > 0.05). The relationships between the immune responses and dietary FPBM levels were, lysozyme activity (y = −0.0229x^2^ + 0.5396x + 53.515, *R*^2^ = 0.9086, optimal dose = 11.76%), IgM (y = −0.0018x^2^ + 0.049x + 4.1333, *R*^2^ = 0.8607, optimal dose = 13.61%), lysozyme activity (y = −0.0067x^2^ + 0.15x + 31.087, *R*^2^ = 0.893, optimal dose = 13.61%), and NBT (y = −5E−05x^2^ + 0.0014x + 0.2355, *R*^2^ = 0.7548, optimal dose = 14%) (**Table 7**).

**Table 5:** Immune responses of Nile tilapia fed FPBM diets for 60 days^*^.

**Item**	**FPBM (%)**					***P* value**
	**0**	**10**	**20**	**30**	**40**	
Lysozyme activity (unit/ml)	30.5 ± 0.75^bc^	33.0 ± 1.03^c^	31.1 ± 0.55^bc^	28.8 ± 0.59^ab^	26.8 ± 0.88^a^	0.03
Phagocytic activity (%)	51.9 ± 0.95^b^	60.2 ± 2.59^c^	53.5 ± 0.94^b^	47.6 ± 0.60^b^	39.5 ± 1.25^a^	0.005
Phagocytic index	2.12 ± 0.05^a^	2.60 ± 0.03^c^	2.52 ± 0.05^c^	2.33 ± 0.06^b^	2.12 ± 0.05^a^	0.032
IgM (mg/dl)	4.03 ± 0.03^ab^	4.60 ± 0.12^b^	4.50 ± 0.12^b^	3.63 ± 0.19^a^	3.30 ± 0.25^a^	0.021
Blood total protein (g/dl)	3.90 ± 0.06	4.03 ± 0.03	4.07 ± 0.07	3.93 ± 0.07	3.90 ± 0.06	0.06
NBT (OD at 630 nm)	0.23 ± 0.01^ab^	0.26 ± 0.01^b^	0.24 ± 0.01^ab^	0.22 ± 0.01^a^	0.21 ± 0.01^a^	0.042
Bactericidal activity (%)	42.6 ± 0.36^b^	45.3 ± 0.62^b^	43.1 ± 0.64^b^	41.7 ± 0.60^b^	29.6 ± 2.71^a^	0.035

* *Values expressed as means ± SE (n = 3). Different superscript letters indicate significant differences for each pairwise comparison between treatments*.

### Oxidative Status

The blood oxidative parameters (SOD, CAT, GPx, and MDA) of tilapia fed FPBM were displayed in Table 6. The group fed the FPBM30 diet showed significantly higher GPx than the control group (*P* <0.05). The relationship between GPx and dietary FPBM levels is y = −0.0046x^2^ + 0.2094x + 26.038, *R*^2^ = 0.8513, optimal dose = 22.76% (Table 7).

**Table 6:** Oxidative status of Nile tilapia fed FPBM diets for 60 days^*^.

**Item**	**FPBM (%)**					***P* value**
	**0**	**10**	**20**	**30**	**40**	
SOD (IU/L)	29.3 ± 0.65	30.5 ± 0.35	30.8 ± 0.40	30.3 ± 0.36	29.3 ± 0.46	0.06
CAT (IU/L)	29.1 ± 0.48	30.8 ± 0.47	31.0 ± 0.58	30.4 ± 0.49	29.2 ± 1.11	0.12
GPx (IU/L)	26.1 ± 0.60^a^	27.5 ± 0.79^ab^	28.0 ± 1.00^ab^	28.7 ± 0.27^b^	26.7 ± 0.56^ab^	0.021
MDA (nmol/ml)	17.0 ± 0.58	16.3 ± 0.88	16.6 ± 0.31	17.2 ± 0.37	18.3 ± 1.36	0.14

* *Values expressed as means ± SE (n = 3). Different superscript letters indicate significant differences for each pairwise comparison between treatments*.

**Table 7:** Regression analysis based on different parameters of Nile tilapia fed FPBM diets for 60 days^*^.

**Indexes**	**Regression equation**	***R*^**2**^**	***P* value**	**Optimal dose (%)**
FBW (g)	y = −0.0108x^2^ + 0.4121x + 41.113	0.9699	0.003	19.08
WG (%)	y = −0.1047x^2^ + 3.9733x + 287.63	0.9795	0.003	18.79
SGR (% IBW/day)	y = −0.0004x^2^ + 0.0164x + 2.2576	0.9829	0.002	20.5
FI (g/fish/60 day)	y = −0.0042x^2^ + 0.1244x + 41.301	0.9647	0.005	14.81
FCR (g FI/g WG)	y = 0.0003x^2^ – 0.0127x + 1.3555	0.9049	0.04	21.17
Amylase (unit/mg)	y = −0.0108x^2^ + 0.377x + 31.913	0.8208	0.001	17.45
Lipase (unit/mg)	y = −0.0318x^2^ + 1.1851x + 23.374	0.8936	0.04	18.63
Protease (unit/mg)	y = −0.0069x^2^ + 0.3469x + 29.239	0.9711	0.001	25.14
Lysozyme activity (unit/ml)	y = −0.0067x^2^ + 0.15x + 31.087	0.893	0.03	11.19
Phagocytic activity (%)	y = −0.0229x^2^ + 0.5396x + 53.515	0.9086	0.005	11.76
Phagocytic index	y = −0.0011x^2^ + 0.0396x + 2.1834	0.8365	0.032	17.9
IgM (mg/ml)	y = −0.0018x^2^ + 0.049x + 4.1333	0.8607	0.021	13.61
NBT (OD at 630 nm)	y = −5E−05x^2^ + 0.0014x + 0.2355	0.7548	0.042	14
Bactericidal activity (%)	y = −0.0206x^2^ + 0.5275x + 42.285	0.9521	0.035	12.8
GPx (IU/L)	y = −0.0046x^2^ + 0.2094x + 26.038	0.8513	0.021	22.76

* *The parameters showed significant differences (P < 0.05) are selected to be represented in the table*.

## Discussion

### Tilapia Growth Performance

The intensification of using a blend of fermented animal proteins has been made it necessary to formulate the most cost-effective balanced feed with the sound nutrition. Most of the studies concluded that the growth performance retardation was detected in the fish fed 75% of FM substitution, irrespective of the inclusion of PBM (10, 12, 13, 27, 41–46). In contrast, the results of the present study revealed that FPBM was successfully included up to 40% in the diet and did not impair Nile tilapia feed efficiency (FI and FCR) and growth performance. Moreover, by including FPBM at 20%, fish obtained better growth performance compared to control. Similarly, Juvenile *L. rohita* tolerates up to 75% replacement of FM with FPBM (16).

In this study, the survival rate of fish fed diets with FPBM remained high during the trial. This agrees with numerous studies that tested FPBM protein in the diets of *L. rohita* (16) and Indian major carp (20). The obtained results in the current study revealed that growth parameters of fish were increased at 19.1–25.14% levels of FPBM. However, fish fed the diet with high level of FPBM (40%) showed reduced growth performance and feed utilization. In the case of high inclusion level (40%), the low digestive enzyme activity and feed palatability are among the factors, which could reduce the feed efficiency and accordingly the growth of fish (46). Fuertes et al. (6) reported that low feed intake and low feed efficiency ratio (increased FCR) resulted from high PBM inclusion levels in crayfish (*Pacifastacus leniusculus*). The fermentation process can increase the apparent digestibility of nutrients in animals (5, 47). The improved feed efficiency in FPBM20 level could be attributed to the level of peptides which increased as a result of the fermentation process by enzymatic degradation, where small-size peptides resulted from protein fractions degradation. These small-size peptides can be absorbed more efficiently by cells of the intestine (48).

The lowest FCR was observed for fish fed the diet with FPBM where 20% was included and did not significantly different from those obtained for the groups fed FPBM10 and FPBM30 levels, suggesting beneficial role of using *S. cerevisiae* to ferment PBM. However, the highest inclusion level of FPBM significantly (*P* < 0.05) increased FCR similar to the control group. It also indicates that the inclusion of high level of FPBM (40%) would not produce any stress or negative impact on the feed efficiency of Nile tilapia.

### Digestive Enzymes Activity

The digestive enzymes activity can increase the feed utilization of diet in the fish intestine (49). FPBM in the experimental diets increased the amylase, lipase, and protease activities. Numerically, the highest amylase and lipase values were in FPBM10 and FPBM20 groups, while the protease activity was increased up to 40% of FPBM inclusion. However, the poorest values were noticed with the groups fed with Con. It is believed that the production of metabolites like enzymes, antimicrobial substances is not only higher in quantity during fermentation and would also be more beneficial in enhancing the nutritive values of ingredients (5, 50, 51). High levels of inclusion may weaken the digestion and use of feed by affecting digestive enzyme activity. The decreased amylase and lipase activities in fish fed FPBM40 in the current study compared to those fed the lowest levels due to the high levels of fibers and non-digestible nutrients. The effect of experimental diets, in relating to FPBM inclusion, on the activity of digestive enzymes is scarce. However, it is a necessity for further investigation in digestive enzymes at gene transcriptional level.

### Immune Responses

Fermentation of PBM resulted in improved immune components measured in the current study. In this study, fish fed FPBM30 diet did not impair the measured immune responses “e.g., activities of lysozyme, phagocytosis, and bactericidal,” while the IgM and NBT levels increased in fish fed FPBM10 and FPBM20 diets. Lysozyme is a proteolytic enzyme with the double role of killing bacteria by disrupting their cell wall and triggering other immune responses such as the complement system and phagocytic cells (52). Lysozyme plays thus an important role in the innate immune system and higher lysozyme levels have been related to better fish immune status. Phagocytosis and bactericidal activity are vital cellular immune responses that can protect fish from pathogens through discovering the existing of infectious pathogens (53, 54). The lysozyme activity increased in Con, FPBM10, and FPBM20 groups and started to decrease by feeding more than 30% FPBM. A similar improvement in blood phagocytic and bactericidal activities were observed in the current study by feeding Con, FPBM10, FPBM20, and FPBM30 diets when compared to fish fed FPBM40 diet.

Natural antibodies such as immunoglobulins (IgM) play a key role in both innate and adaptative immunity, producing specific antibody responses against various antigens (55). The activity of respiratory bursts (NBT) is also necessary for the assessment of the fish general health (36, 56). Respiratory burst has a critical role in fish immune system by increasing the levels of phagocytes which can release the high levels of ROS in fish cells (54, 57). In this study, we observed that FPBM10 and FPBM20 diets increased the serum IgM levels, while NBT was significantly increased by feeding FPBM10 diet compared with FPBM30 and FPBM40 groups in Nile tilapia.

The measured immune responses (lysozyme, phagocytosis, bactericidal, and NBT activities as well as IgM level) were decreased in fish the highest inclusion level of FPBM (FPBM40), which could be as a result from impaired immunity and increased oxidative stress caused by low protein quality and higher fiber fractions in FPBM40 diet, agreeing with previous studies on the importance of optimum animal protein source used in diet formulation (5, 16, 20).

### Oxidative Status

The oxidative emphasis normally happens when the creation and elimination of free radicals (ROS) are unbalanced since the oxidative damage of cultured species is directly related to the quality of diet (58). MDA is a product of lipid peroxides and high levels of ROS, which can cause damage to cell's DNA, protein, and cytoplasm (59). In this experiment, fish fed FPBM30 diet showed improved GPx without affecting the MDA level indicating improved antioxidant activity which can be attributed to the presence of bioactive compounds that may improve the antioxidant enzyme activity and regulate the ROS production (60). As an antioxidant, GPx mainly exists in cell metabolism, playing a protective role in cells by the disproportionation of toxic ROS to inactive hydrogen peroxide and oxygen molecules (61).

## Conclusion

In conclusion, appropriate dietary FPBM level improved the general performances of Nile tilapia. Based on the measured parameters, the recommended inclusion levels of dietary FPBM for Nile tilapia is 11.17–25.14% diet. Fermented PBM shown to have an enhanced nutritional status in terms of higher protein and essential amino acids in comparison to the respective untreated ones. Therefore, it is believed that FPBM would be more effective rather using PBM in tilapia feed, with a great potential in reducing the pressure on FM.

## Data Availability Statement

All datasets generated for this study are included in the article/supplementary material.

## Ethics Statement

The experiment was conducted in accordance with the guideline of Kafrelshiekh University, Egypt. All animal protocols were approved by the Institutional Animal Care and Use Committee of Faculty of Agriculture, Kafrelsheikh University.

## Author Contributions

All persons listed as authors have read, contributed to preparing the manuscript as given below: MD and MM carried out fish maintenance and sample collection. FM, AS, AA, SF, and HA carried out the experimental design, required analysis, and statistical analyses. KA-G, SM, and FA-M carried out data interpretation.

### Conflict of Interest

The authors declare that the research was conducted in the absence of any commercial or financial relationships that could be construed as a potential conflict of interest.
